# Estimating the association between Facebook adoption and well-being in 72 countries

**DOI:** 10.1098/rsos.221451

**Published:** 2023-08-09

**Authors:** Matti Vuorre, Andrew K. Przybylski

**Affiliations:** ^1^ Oxford Internet Institute, University of Oxford, Oxford OX1 3JS, UK; ^2^ Tilburg School of Social and Behavioral Sciences, Tilburg University, Tilburg, The Netherlands

**Keywords:** well-being, social media, life satisfaction

## Abstract

Social media's potential effects on well-being have received considerable research interest, but much of past work is hampered by an exclusive focus on demographics in the Global North and inaccurate self-reports of social media engagement. We describe associations linking 72 countries' Facebook adoption to the well-being of 946 798 individuals from 2008 to 2019. We found no evidence suggesting that the global penetration of social media is associated with widespread psychological harm: Facebook adoption predicted life satisfaction and positive experiences positively, and negative experiences negatively, both between countries and within countries over time. Nevertheless, the observed associations were small and did not reach a conventional 97.5% one-sided credibility threshold in all cases. Facebook adoption predicted aspects of well-being more positively for younger individuals, but country-specific results were mixed. To move beyond studying aggregates and to better understand social media's roles in people's lives, and their potential causal effects, we need more transparent collaborative research between independent scientists and the technology industry.

## 1. Introduction

The ways in which people use technology for most domains in life has changed dramatically since the mass introduction of the Internet in the 1990s, and the subsequent technologies facilitated by it. Most prominently, the popularization of modern social media platforms circa 2008 precipitated widespread changes to human activities via features such as marketplaces, personalized news feeds, photo sharing, live streaming and other features that the ‘metaverse’ now promises to build on. The first social media with broad adoption, MySpace (launched 2003), saw 115 million users in 2008—the year in which it was replaced as the leading platform by Facebook (2004). In 2022 (Q1), Facebook reported 2.94 billion monthly active users [1], or about one third of the global population. Along with social media's global penetration, debate surrounding their potential effects on individual and collective well-being has intensified.

Although reports of negative psychological outcomes associated with social media are common in academic and popular writing [2,3], empirical evidence for harms is, on balance, more speculative than conclusive [4–6]. Recent results on the associations between social media use and well-being are mixed and depend on arbitrary analytic choices [7]. Other studies have reported that there have been few if any changes in associations linking technology use to mental health in this period of social media's global adoption [8]. A general lack of validated measures, poorly specified causal models, and inadequate data have yielded a large number of low-quality studies [9,10]. Furthermore, because nearly all investigations have focused on samples from the Global North [11], we have next to no idea of how the wider adoption of social media platforms relates to psychological well-being across the world.

Here, we took a different approach to understanding how social media might relate to well-being. Instead of focusing on individual-level data, we focused on trends and associations at the broad level of demographic groups within countries and over time. This broad approach allowed us to investigate a dramatically broader scope of geographies and demographies than previous attempts [11].

We conducted a descriptive study that linked data tracking Facebook's global adoption with three indicators of well-being. We examined 72 countries' *per capita* active Facebook users in two age brackets (13–34 and 35+ years) as predictors of life satisfaction (LS), negative (NE) and positive psychological experiences (PE) at the level of years spanning 2008 to 2019. The well-being data represented 946 798 individuals’ responses from the nationally representative Gallup World Poll Survey [12].

We joined these unique datasets to conduct a descriptive study to answer three basic yet important questions. First, to what extent is Facebook adoption associated with well-being? Second, do these associations differ by age or sex [7,13,14]. And finally, how might these associations have differed between countries? In addition, we were interested in whether the intensity of use might make a difference, and therefore conducted our analyses separately for daily active users and monthly active users. As a supplementary analysis, we replicated and present these analyses on meta-analytic mental health outcomes in an appendix. Due to the exploratory and descriptive nature of our study, we did not have *a priori* hypotheses about the directions or magnitudes of the potential associations.

## 2. Methods

### 2.1. Facebook data

We studied two metrics of Facebook adoption at the level of years and countries: daily (DAU) and monthly active users (MAU), from 2008 to 2019 for 72 countries (figure 2). DAU indicates the number of individuals who used Facebook or Messenger on a given day, and accounts for any use of either product (e.g. a login to Facebook). The Facebook definition of DAU was ‘A registered and logged-in Facebook user who visited Facebook through our website or a mobile device, or used our Messenger application (and was also a registered Facebook user), on a given day’. For MAU, it was ‘A registered and logged-in Facebook user who visited Facebook through our website or a mobile device, or used our Messenger application (and was also registered Facebook user), in the last 30 days as of the date of measurement’.

To aggregate DAU to the level of years and countries as analysed here, Facebook used the mean DAU in the time period from 1 June to 31 August for each year and country. Values greater than 10 000 000 were rounded to three significant digits, and values lower than 10 000 000 to the nearest 10 000. MAU was calculated identically, but accounts for any use within a one month window.

Facebook calculates DAU and MAU estimates separately for individuals aged 13–34 and 35+. User age is determined based on Facebook profile information, which can be inaccurate (e.g. young users reporting an older age). Accordingly, Facebook has trimmed 0.008% of total MAU to exclude accounts with unrealistic or non-reported ages. Facebook chose the 13–34 and 35+ age categories in order to maximize the accuracy of the data. Nevertheless, the lower age category includes groups typically defined as adolescents and young adults [15].

In personal communication, Facebook representatives explained the selection of countries as ‘The countries provided were selected based upon geographic and cultural diversity and criteria related to data quality, including that geographic and age attribution error is believed to be relatively small’. A given user's country is determined based on a number of factors, including the user's IP address and self-reported location.

Although accurately captured, these numbers are not perfect indicators of actual user numbers because of possible duplicate and false accounts. Facebook estimates those accounts to account for 11% and 5% of global MAUs, respectively, and that the former may be more likely in developing regions. Because internal criteria and methodology for determining duplicate and false accounts can change over time, estimates of MAU and DAU can also change.

Then, to make DAU and MAU comparable across countries and age groups, we converted them to proportions of each country's and age group's yearly population sizes using population data from the United Nations Department of Economic and Social affairs (https://population.un.org/wpp/Download/Standard/Population/). Thus, each value of DAU (MAU) below refers to the proportion of population in a given country in a given year that used Facebook or Messenger on a daily (monthly) basis. No observations were removed for analyses.

The Facebook adoption data were made available to us on Facebook's Open Research Tool platform. Other researchers can contact Facebook (ccobb@fb.com) to access the dataset.

### 2.2. Well-being

Gallup World Poll (GWP) is a nationally representative annual survey of 1000 civilian, non-institutionalized individuals aged 15 years or older from 164 countries conducted since 2005. The surveys are conducted face-to-face or on the phone in the respondents' native language and by local interviewers (for details, see [12]). We studied positive (PE) and negative experiences (NE), and life satisfaction. PE and NE measure respondents’ experienced well-being on the day before the survey with five items each. For PE, these items are: ‘Did you feel well-rested yesterday?’, ‘Were you treated with respect all day yesterday?’, ‘Did you smile or laugh a lot yesterday?’, ‘Did you learn or do something interesting yesterday?’, and ‘(Did you experience the following feelings during a lot of the day yesterday?) How about enjoyment?’. The NE items are responses to ‘Did you experience the following feelings during a lot of the day yesterday?’ for ‘physical pain’, ‘worry’, ‘sadness’, ‘stress’ and ‘anger’. For analyses we used the means of these scales.

Prior studies using the NE and PE scales have found them to display acceptable validity and measurement invariance, and that response-style differences across world regions are small [16]. In addition, Gallup claims that its questions are developed ‘using a global network of research and political scientists [The Brookings Institution, World Bank, USAID, United Nations, Daniel Kahneman, Ed Diener, Deepak Chopra, Richard Florida, John Helliwell, Jeffrey Sachs and Arthur Stone] who understand key issues concerning question development and construction and data gathering’ [17, p. 5]. Although not extensively validated, we believe these items and scales to be uniquely valuable for our goals due to their extensive scope across time, geography and demographics.

Life satisfaction in the moment was measured with one 11-step Likert item, the Cantril Self Anchoring Scale (or ‘Cantril ladder’): ‘Please imagine a ladder, with steps numbered from 0 at the bottom to 10 at the top. The top of the ladder represents the best possible life for you and the bottom of the ladder represents the worst possible life for you. On which step of the ladder would you say you personally feel you stand at this time?’ [18]. In addition to inclusion in the data, we were motivated to use this scale due to its widespread use [19], and because of prior work establishing its reliability and validity [20].

For analyses, we scaled these variables to percentages, and aggregated the 946 798 individuals' data to means and standard errors for each country, year, sex and age (15–34 and 35+) combination (3136 cells).

### 2.3. Data analysis

We examined the association between Facebook adoption and well-being through Bayesian hierarchical regression models, estimated separately for DAU and MAU, and each of the three well-being outcomes. We regressed the outcome *y* on time (decades, centred on 2014), within-country centred DAU or MAU (in separate models), age, sex, all the interactions except time by DAU/MAU, and the between-country centred DAU/MAU. We allowed all coefficients to vary randomly across the 72 countries, except the between-country predictor. Because we aggregated the outcomes, we included standard errors of the outcome in the model to account for the varying group sizes and resulting uncertainties in the modelled data. Formally, we specified the model as

$$\begin{array}{rl} y_{i}∼\; & \mathrm{Normal}\left(\mu_{i},\sqrt{\sigma^{2}+{\mathrm{se}}_{i}^{2}}\right),\\ \mu_{i}=\; & \alpha_{0}+\beta_{0\mathrm{country}[i]}+(\alpha_{1}+\beta_{1\mathrm{country}[i]}){\mathrm{Sex}}_{i}\\ & +(\alpha_{2}+\beta_{2\mathrm{country}[i]}){\mathrm{Age}}_{i}+(\alpha_{3}+\beta_{3\mathrm{country}[i]}){\mathrm{Time}}_{i}\\ & +(\alpha_{4}+\beta_{4\mathrm{country}[i]}){\mathrm{DAU}}_{i}^{\mathrm{CW}}+(\alpha_{5}+\beta_{5\mathrm{country}[i]}){\mathrm{Sex}}_{i}\times {\mathrm{Time}}_{i}\\ & +(\alpha_{6}+\beta_{6\mathrm{country}[i]}){\mathrm{Age}}_{i}\times {\mathrm{Time}}_{i}+(\alpha_{7}+\beta_{7\mathrm{country}[i]}){\mathrm{Sex}}_{i}\times {\mathrm{Age}}_{i}\\ & +(\alpha_{8}+\beta_{8\mathrm{country}[i]}){\mathrm{Sex}}_{i}\times {\mathrm{Age}}_{i}\times {\mathrm{Time}}_{i}+(\alpha_{9}+\beta_{9\mathrm{country}[i]}){\mathrm{Sex}}_{i}\\ & \times {\mathrm{DAU}}_{i}^{\mathrm{CW}}+(\alpha_{10}+\beta_{10\mathrm{country}[i]}){\mathrm{Age}}_{i}\times {\mathrm{DAU}}_{i}^{\mathrm{CW}}+(\alpha_{11}+\beta_{11\mathrm{country}[i]}){\mathrm{Sex}}_{i}\\ & \times {\mathrm{Age}}_{i}\times {\mathrm{DAU}}_{i}^{\mathrm{CW}}+\alpha_{12}{\mathrm{DAU}}_{i}^{\mathrm{CB}},\\ & \beta \tilde{\mathrm{MVN}}(0,\Sigma ), \end{array}$$

where $y_{i}$ is the outcome (e.g. life satisfaction) on row *i*, ${\mathrm{se}}_{i}$ its standard error, α are the population-level coefficients, $\beta_{0,\ldots ,11\mathrm{country}[i]}$ are the country-specific coefficients for the country indicated on row *i*, ${\mathrm{DAU}}_{i}^{\mathrm{CW}}$ the within-country centred year-aggregated daily (or monthly) active Facebook users, and ${\mathrm{DAU}}_{i}^{\mathrm{CB}}$ the between-country centred DAU (or MAU).

We conducted all data analyses with the R language for statistical computing [21] and estimated the models using Stan's Hamiltonian Monte Carlo sampling via the brms R package [22,23]. We used default noninformative priors, 4 HMC chains with 4000 iterations and first 2000 as warmup for 8000 total iterations; we report all parameters with their posterior means and 95% credible intervals (posterior 2.5 and 97.5 percentiles; CI), and other posterior probabilities as indicated in text.

## 3. Results

**Figure 1.**
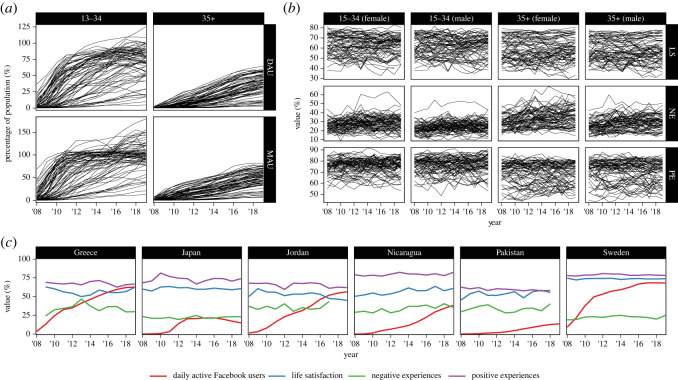
(*a*) 72 countries' daily (top) and monthly (bottom) active Facebook users in two age brackets from 2008 to 2019. (Percentages may exceed 100% due to inaccurate estimation of either DAU or population size.) (*b*) The same 72 countries' mean responses to three well-being scales in the Gallup World Poll from 2008 to 2019, separated by age category and sex. LS: life satisfaction; NE: negative experiences; PE: positive experiences. (*c*) Country-year means of daily active Facebook users and three well-being metrics from 2008 to 2019 for a random sample of countries.

Facebook adoption increased markedly from 2008, when the mean *per capita* DAU across these 72 countries was 4% (ages 13–34) and 0% (35+), to 70% (13–34) and 37% (35+; figure 1*a*) in 2019. The mean MAUs in 2019 were greater at 98% (13–34) and 49% (35+). During this period Facebook adoption by younger individuals reached near 100% in many countries, but not for older individuals. At the same time, we did not observe correspondingly large and uniform changes across measures of well-being; life satisfaction had remained relatively stable, whereas both negative and positive experiences had slightly increased (figure 1*b*) [24]. We show these data in more detail in figure 1*c* to allow a visual comparison of Facebook adoption and well-being trends within those nations.

We then focused on our first question: how Facebook adoption relates to well-being in the average country and demographic. We first examined whether and how the relative standing of countries on their average Facebook adoption predicted well-being (between-country associations, $\alpha_{12}$). We found that countries with greater average daily active Facebook users (DAU) had higher levels of life satisfaction and positive experiences, and lower levels of negative experiences, than countries with lower DAU (table 1; between countries). However, there are large and important differences between countries in factors that might underlie differences in both social media adoption and well-being, such as socioeconomic conditions [25]. While descriptively informative, these associations are therefore likely to indicate between-country confounding factors.

**Table 1.** Average Facebook adoption and well-being associations. Numbers indicate posterior means, [95% CIs], and (posterior probabilities of direction).

predictor	outcome	between countries	within country
DAU	life satisfaction	0.42 [0.32, 0.52] (>99.9%)	0.01 [−0.02, 0.04] (82.2%)
	negative experiences	−0.09 [−0.19, 0.01] (96.8%)	−0.03 [−0.07, 0.00] (97.4%)
	positive experiences	0.11 [0.02, 0.19] (99.2%)	0.03 [0.00, 0.06] (98.4%)
MAU	life satisfaction	0.33 [0.24, 0.41] (>99.9%)	0.01 [−0.01, 0.03] (83.8%)
	negative experiences	−0.06 [−0.13, 0.01] (94.8%)	0.00 [−0.02, 0.03] (53.1%)
	positive experiences	0.08 [0.02, 0.15] (99.2%)	0.04 [0.01, 0.06] (99.8%)

Variations in such confounding factors are likely to be significantly smaller within countries but over time in a 12-year period. We therefore next focused on the model's within-country associations ($\alpha_{4}$). They measure the extent to which Facebook adoption in a given country predicted well-being in that country, adjusting for temporal trends in the country's well-being outcome. For the average country and across age and sex, we found that within-country increases in DAU predicted greater levels of life satisfaction and positive experiences, and lower levels of negative experiences, although only the positive experience association's 95% CI excluded zero (table 1; within country).

However, these results are qualified because a focus on daily active users could miss those who use Facebook less regularly. To test this possibility, we also conducted our analyses using monthly active Facebook users (MAU) as the predictor. Both the between- and within-country associations linking MAU and well-being were very similar to those linking DAU and well-being, although in general of smaller magnitude. Moreover, the within-country link between MAU and negative experiences' credibility interval was narrowly centred on zero, indicating relative certainty that the association is practically equivalent to zero.

In addition to sign tests, we quantified evidence for the associations to be greater in magnitude to a 0.01% change as a function of 1% increase in MAU/DAU [26]. DAU predicted negative and positive experiences in excess of 0.01% with 91.6% and 93.0% probability, respectively, and MAU predicted positive experiences in excess of 0.01% with 98.2% probability. In sum, then, for the average country in our sample, Facebook adoption positively predicted well-being. Nevertheless, our certainties in the directions of these associations were not great, and the magnitudes of these associations were small: a 1% increase in DAU predicted a 0.03 [0.00, 0.06] (98.4%) increase in positive experiences for the average country. While these associations indicate relations within countries and adjust for confounders that vary linearly with time by including time as a predictor, they are still susceptible to confounders and do not indicate causal relations. Rather, this association describes that all else being equal, years with greater Facebook adoption tended to be those with greater levels of positive experiences for the average country.

**Figure 2.**
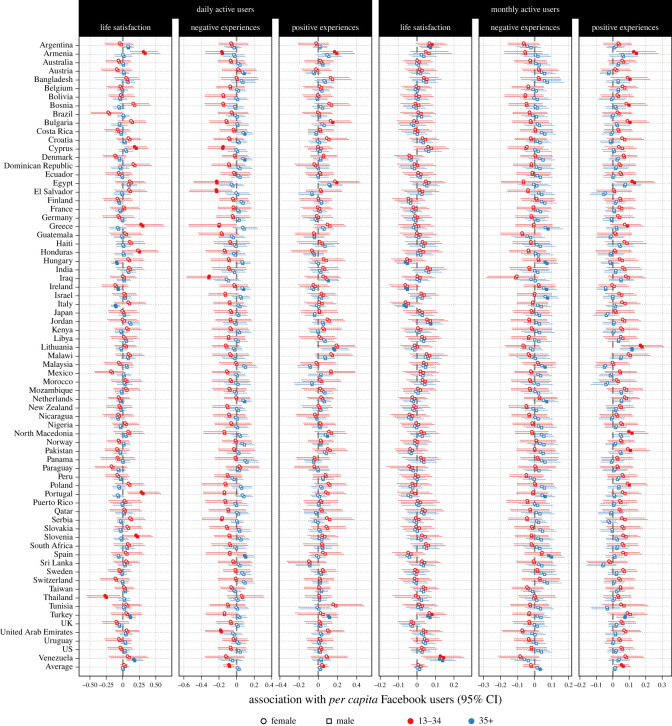
Within-country associations between daily (left; DAU) and monthly (right; MAU) active Facebook users and life satisfaction (LS), negative experiences (NE), and positive experiences (PE). Units indicate percentage change in outcome as a function of percentage increase in within-country centred DAU or MAU. Filled points indicate estimates whose 95% CI excludes zero.

We then turned to our second question and assessed whether within-country associations linking Facebook adoption to well-being differed between age and sex. DAU predicted negative experiences more negatively, and MAU positive experiences more positively, for the younger age group than for the older (0.05 [0.02, 0.08] (greater than 99.9%), −0.02 [−0.04, 0.00] (98.5%)). Other average age differences were not credibly different from zero at the 95% level. The association between DAU and MAU and well-being was more positive for males than it was for females, across all well-being measures, but the differences were not credibly different from zero. We display the average age- and sex-specific associations in the bottom row of figure 2. Overall, these results indicated that the association between Facebook adoption and well-being was more positive for younger individuals, particularly for negative and positive experiences, and that sex-based differences were much smaller in magnitude and not credibly different from zero.

However, while informative aggregates, these results do not describe associations between Facebook adoption and well-being for any individual country, but rather for the average country in this sample of 72 countries. To answer our third question, we computed country-specific estimates for each sex and age group (figure 2). For life satisfaction, 2 countries had credibly positive, and 0 countries negative, average associations with DAU. For negative experiences, 0 countries had credibly positive and 3 had negative average association. Four countries had a credibly positive average association but 0 had a negative association between DAU and positive experiences. The corresponding results but with MAU as the predictor were very similar. Overall, the country-level estimates did not lend support to the idea of widespread negative associations between social media adoption and psychological well-being. Nevertheless, we note that with limited data, it is difficult to determine these associations with great confidence for any given country.

## 4. Discussion

It is widely accepted that social media and the Internet more broadly have changed how humans socialize, organize, and seek leisure, but it is not obvious or necessary that their wide adoption has influenced psychological well-being. In this descriptive study we used the broadest data available to describe how two measures of Facebook adoption relate to three well-being outcomes across 72 countries over a 12-year period. We found generally positive associations between country-level Facebook adoption and well-being which were partially qualified by demographics and not uniform across countries. We did not find evidence that increased social media adoption is consistently negatively associated with well-being.

Overall, a country's *per capita* daily active Facebook users predicted that nation's demography-aggregated levels of positive experiences positively, and negative experiences negatively. In addition, the associations between countries were similar, but the uncertainty cutoff of 97.5% for posterior probabilities of direction was strictly only met for positive experiences (table 1). Associations between Facebook adoption and life satisfaction were less certain within countries, but stronger when comparing countries to each other. While these descriptive results do not speak to causal effects, they align with other findings suggesting that technology use has not become increasingly associated with negative psychological outcomes over time [8], and that the increased adoption of Internet technologies in general is not, overall, associated with widespread psychological harms [24]. We also found that Facebook adoption predicted young demographics' positive well-being more strongly than it did older demographics’, and that sex differences in this dataset were very small and not credibly different from zero. These demography-based differences, and lack therein, were notable in light of previous literature that has reported young girls to be more at-risk of screen- and technology-based effects than young males (e.g. [27]; but see [28]). However, those studies focused on younger individuals (from 10 to 15 years old), which likely partly explains the different findings.

We also conducted these analyses using two different metrics of Facebook adoption: daily active users and monthly active users. It was important to study both, as they indicate different types of engagement with the platform, and it is possible that meaningful associations might emerge only for more intense types of engagement (daily active users). We found that the results were, by and large, in agreement. In addition, in appendix A we studied Facebook adoption in relation to meta-analytic estimates of country-level rates of anxiety, depression and self-harm [29]. Those results did not indicate strong evidence either for or against associations. Instead, they reinforce the position that better data, on both Facebook adoption and global mental health, are urgently needed to better understand how they might relate.

In this study, we aimed to accurately describe how broad demographic groups' trends in well-being are associated with Facebook adoption at the level of individual countries. That is, we did not investigate whether, for example, days during which individuals use more social media are also days in which they report better or worse well-being. Instead, our investigation was focused on broader trends and associations. For example, social media use might have indirect relations to well-being among groups of individuals, such that even if an individual abstains from use, their peer group might be affected and transmit any negative effects via social contagion.

For the same reason, there are likely to be large differences within countries in our aggregated data in the degree of social media adoption and well-being that we could not address. Our analyses also cannot address qualitative dimensions of individuals’ social media use thought to moderate associations between social media use and well-being, such as whether the use is active or passive, or whether user motivations are goal-directed or mere procrastination [30,31]. In addition, our descriptive analyses cannot and do not rule out the possibility of causal effects, either negative or positive, between social media use and well-being. More fine-grained data needed to demonstrate causal relations, or lack thereof, more conclusively either do not exist or are not available to independent scientists. We also did not make attempts at finding a socially or geographically representative sample of nations to study, but rather used data from countries that Facebook determined to have the most accurate data about adoption and demographics. It is therefore possible that these results would not generalize beyond the sample of 72 nations we studied.

We also highlight the fact that while Facebook adoption remains the overall dominant social media platform, our results do not necessarily generalize across different platforms. For instance, in the United States, 13- to 17-year-olds are more likely to use TikTok, Instagram and Snapchat than Facebook, so the user base of Facebook now consists of relatively more older individuals [32]. In addition to the demographic shifts between social media platforms, the platforms themselves change over time and their associations with well-being might therefore not be consistent over time or different countries [8]. Moreover, studying a single platform cannot provide a complete picture of the overall associations that social media as a whole might have with well-being, because different platforms are used by different people and for different purposes, all of which might serve to moderate any potential associations.

If we are to move past description, the goal of this study, to prediction or evidence-based intervention, independent scientists and online platforms will need to collaborate in new, transparent ways. As it stands now, only a handful of scientists working in the technology industry have the data required to advance this line of inquiry. If we are to understand and improve well-being in the digital age, this must change.

## Figures and Tables

**Figure 3.**
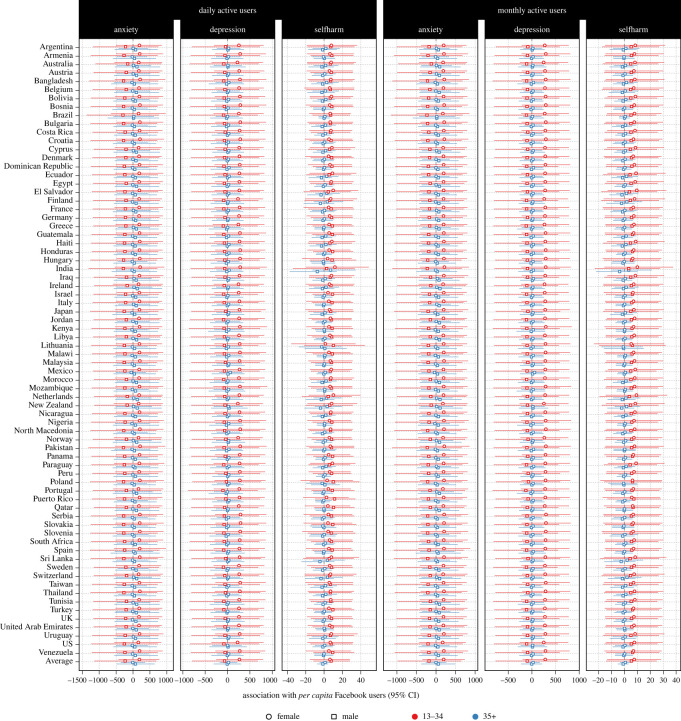
Within-country associations between daily (left; DAU) and monthly (right; MAU) active Facebook users and three mental health outcomes. Units indicate change in outcome rate (per 100 000 individuals) as a function of percentage increase in within-country centred DAU or MAU. Filled points indicate estimates whose 95% CI excludes zero.

## Data Availability

Both datasets (Facebook and Gallup) are proprietary and we therefore could not share them with this paper. Our analytic code, along with synthetic datasets, is available at http://dx.doi.org/10.5281/zenodo.7086277 [33]. This study was not preregistered. Researchers can contact Facebook (ccobb@fb.com) to reproduce our analyses with the actual Facebook adoption dataset. The Gallup well-being data are available online to subscribing institutions.

## Appendix A. Meta-analytic mental health outcomes

The main focus of this study was on well-being, as operationalized in the three Gallup World Poll measures. In addition, we replicated the analyses in this supplement, but used meta-analytic rates (per 100 000 individuals) of anxiety disorders (ICD10 F40-F44.9, F93-F93.2), depressive disorders (ICD10 F32-F33.9, F34.1), and self-harm (ICD10 X60-X64.9, X66-X84.9, Y87.0), as estimated by the Global Burden of Disease 2019 (GBD) study [29,34] as the outcomes. The prevalence rate estimates are based on meta-analyses of 19 773 data sources with varying coverage for individual countries; for methodological details, see [29] and especially appendix 1 therein. The prevalence rates are estimated for females and males in 5-year age groups, but we aggregated these to match the age groups in the Facebook dataset.

The GBD estimates are not observed data, but instead reflect the meta-analytic methods of the GBD 2019 study. We have compared the GBD estimates to the CDC's estimates of self-harm in the United States [35], and found that they are likely to deviate in systematic ways from other authoritative information sources. Nevertheless because the GBD provides the most comprehensive dataset of global mental health, studying these estimates can be informative. We analysed the data as above.

We have reported elsewhere, using a superset of the current countries, that rates of anxiety had increased, and rates of depression and self-harm had decreased, for the average country [24]. To answer our first research question, table 2 reports the within- and between-country associations linking the three mental health outcomes with daily and monthly active Facebook users. These data did not indicate strong support for or against the idea that countries' Facebook adoption was associated with those countries’ levels of mental health problems, as reflected in the parameters' wide posterior uncertainty intervals (within-country associations in table 2). Similarly, none of the between-country associations’ credibility intervals excluded zero, but instead indicated great uncertainty about the parameters (between-country associations in table 2).

**Table 2.** Average Facebook adoption and mental health associations*.* Numbers indicate posterior means, [95% CIs], and (posterior probabilities of direction).

predictor	outcome	between countries	within country
DAU	anxiety	361.43 [−861.10, 1 617.43] (71.9%)	3.95 [−333.59, 340.21] (50.9%)
	depression	−286.35 [−1 385.49, 849.93] (70.1%)	52.80 [−200.78, 300.85] (65.2%)
	self-harm	−0.26 [−43.67, 40.85] (50.7%)	3.44 [−5.99, 12.89] (76.3%)
MAU	anxiety	162.88 [−828.54, 1 162.19] (62.3%)	19.18 [−270.23, 312.41] (55.2%)
	depression	−191.71 [−1 090.99, 709.25] (67.2%)	50.69 [−174.87, 266.58] (67.6%)
	self-harm	3.83 [−29.02, 35.49] (59.5%)	3.13 [−5.74, 12.11] (75.2%)

We then examined whether there might have been differences in the associations between the demographic groups, or between countries. We did not find differences between either sex or age groups in the associations linking Facebook adoption to either anxiety, depression or self-harm (all posterior probabilities of direction were smaller than 83%) In a similar vein, any differences between countries were very small (figure 3).

In summary, we extended our main analyses concerning the relationships between country-level Facebook adoption and well-being to the domain of mental health, using openly available meta-analytical estimates of anxiety, depression, and self-harm. Across the six models examining two facets of Facebook adoption and meta-regression estimates of three mental health outcomes, we did not find any associations that were credibly different from zero, or any differences in those associations between demographic groups or countries. However, these results are likely to reflect the invariance in the mental health outcomes combined with the relatively high correlations between the Facebook adoption metrics and time. Therefore, in order to better understand how Facebook adoption might relate to mental health, more detailed data on both Facebook adoption and mental health are urgently required.
